# *Sipjeondaebo-tang* Alleviates Oxidative Stress-Mediated Liver Injury through Activation of the CaMKK2-AMPK Signaling Pathway

**DOI:** 10.1155/2018/8609285

**Published:** 2018-11-06

**Authors:** Sang Mi Park, Sung Woo Kim, Eun Hye Jung, Hae Li Ko, Chae Kwang Im, Jong Rok Lee, Sung Hui Byun, Sae Kwang Ku, Sang Chan Kim, Chung A Park, Kwang Joong Kim, Il Je Cho

**Affiliations:** ^1^Department of Herbal Formulation, College of Korean Medicine, Daegu Haany University, Gyeongsan 38610, Republic of Korea; ^2^Department of Physiology, College of Korean Medicine, Daegu Haany University, Gyeongsan 38610, Republic of Korea; ^3^Department of Pharmaceutical Engineering, College of Bio-Technology, Daegu Haany University, Gyeongsan 38610, Republic of Korea; ^4^Department of Histology and Anatomy, College of Korean Medicine, Daegu Haany University, Gyeongsan 38610, Republic of Korea; ^5^Department of Internal Medicine, College of Korean Medicine, Daegu Haany University, Gyeongsan 38610, Republic of Korea

## Abstract

*Sipjeondaebo-tang* (SDT) is used frequently as a herbal prescription to treat deficiency syndromes in traditional Korean medicine. We investigated the hepatoprotective effects of SDT against oxidative stress and attempted to clarify the underlying molecular mechanisms. SDT pretreatment reduced arachidonic acid (AA) plus iron-mediated cytotoxicity in a concentration-dependent manner and prevented changes in apoptosis-related protein expression. In addition, SDT pretreatment significantly reduced glutathione depletion, hydrogen peroxide production, and mitochondrial dysfunction via treatment with AA plus iron. SDT increased the phosphorylation of AMP-activated protein kinase (AMPK) in accordance with the phosphorylation of Ca^2+^/calmodulin-dependent protein kinase kinase 2 (CaMKK2). Experiments using an AMPK chemical inhibitor (Compound C) or CaMKK2 chemical inhibitor (STO-609) suggested that the CaMKK2-AMPK signaling pathway contributes to SDT-mediated protection of mitochondria and cells. Moreover, administration of SDT for 4 consecutive days to mice significantly reduced the alanine aminotransferase and aspartate aminotransferase activities induced by carbon tetrachloride, and the numbers of degenerated hepatocytes, infiltrated inflammatory cells, nitrotyrosine-positive cells, and 4-hydroxynonenal-positive cells in liver tissue. Therefore, SDT protects hepatocytes from oxidative stress via CaMKK2-dependent AMPK activation and has the therapeutic potential to prevent or treat oxidative stress-related liver injury.

## 1. Introduction

Oxidative stress, caused by an imbalance between reactive oxygen species (ROS) and the antioxidant defense system, plays an essential role in the pathogenesis of various types of liver disease, including hepatitis, steatosis, and fibrosis [1]. Excess ROS generation, i.e., that beyond the adaptive capacity, provokes oxidation of biomolecules in hepatocytes, reduces their cellular function, and promotes cell death. In particular, ROS modifies lipids in the plasma membrane and releases arachidonic acid (AA), a proinflammatory *ω*-6 polyunsaturated fatty acid [2]. AA induces mitochondria-dependent apoptosis of hepatocytes [3]. Moreover, in the presence of iron, AA promotes ROS generation in cells and mitochondria, depletes reduced glutathione (GSH), induces mitochondrial membrane leakage, and accelerates apoptosis of hepatocytes [3, 4].

To cope with oxidative stress, cells regulate the activity of diverse signaling pathways. Among these, AMP-activated protein kinase (AMPK) is an evolutionarily conserved serine/threonine kinase and is regarded as a redox-sensitive master regulator that protects cells from oxidative and metabolic stress [5, 6]. AMPK is a heterotrimeric protein composed of a catalytic subunit (*α* subunit) and two regulatory subunits (*β* and *γ* subunits). Although AMPK isoforms are differentially expressed in different tissues, AMPK activity is regulated by phosphorylation of the catalytic subunit and via allosteric binding of adenosine phosphates to the regulatory subunit [5]. In the liver, AMPK activation halts anabolic processes (e.g., fatty acid, carbohydrate, and protein synthesis) and promotes catabolic processes (e.g., fatty acid oxidation and glucose uptake). Interestingly, AMPK inhibits glycogen synthase-3*β*- (GSK-3*β*-) mediated mitochondrial impairment, facilitates mitochondrial biogenesis via the peroxisome proliferator-activated receptor *γ* coactivator-1*α*, and activates autophagy to remove damaged macromolecules [6]. Because the mitochondrion is an essential subcellular organelle for accelerating oxidative stress, maintaining mitochondrial integrity and homeostasis via AMPK provides a prosurvival signal against oxidative stress. Several medicinal herbs and isolated natural products that protect against oxidative stress via AMPK activation have been identified [4, 7–10].

In traditional Korean, Chinese, and Japanese medicine, no single herb is applied; instead, prescriptions comprising multiple medicinal herbs are frequently used to treat diverse diseases. According to the “king-minister-assistant-ambassador” theory (*Kun-Shin-Jua-Sa* in Korean;* Jun-Chen-Zuo-Shi* in Chinese) in traditional medicine, use of a combination of medicinal herbs can enhance the pharmacological activities of the major acting herbs (i.e., “king drugs”), helps distribute king drugs to the target organ(s), and reduces unwanted side effects of king drugs [11]. Due to the complexity of such herbal prescriptions, studies of their pharmacological effects face challenges and have been limited to date. However, at least in traditional medicine, it is clinically more important and relevant to study herbal formulas rather than single herb extract.* Sipjeondaebo-tang *(SDT;* Shiquandabu* in Chinese,* Juzentaihoto* in Japanese) comprised 12 medicinal herbs and can tonify vital energy (also called “*qi*”) and blood [11]. Thus, SDT has been used to treat lassitude, anemia, and anorexia in traditional Korean medicine [12]. Modern scientific evidence suggests that SDT has a variety of pharmacological activities, including antitumor [13–15], antioxidant [16–18], and neuroprotective [19] activities. In addition, SDT ameliorates the anorexia and hepatotoxicity induced by carbon tetrachloride (CCl_4_)[16], steatosis and fibrosis in response to methionine- and choline-deficient diets [20], steatosis and necroinflammation caused by high fat diet [21], and the hepatocarcinogenesis by diethylnitrosamine [15] in experimental animals. Although the beneficial effects of SDT in diverse type of liver disease might result from its antioxidant and anti-inflammatory activities, the underlying molecular mechanisms are not fully understood. Therefore, we investigate the* in vitro* and* in vivo* hepatoprotective effects of SDT against oxidative stress to elucidate the underlying molecular mechanism.

## 2. Materials and Methods

### 2.1. Reagents

AA, compound C, and STO-609 were purchased from Calbiochem (San Diego, CA, USA). Antibodies directed poly(ADP-ribose)polymerase (PARP), caspase-3, B cell lymphoma- (Bcl-) 2, phosphorylated AMPK*α*, phosphorylated acetyl-CoA carboxylase (ACC), ACC, phosphorylated Ca^2+^/calmodulin-dependent kinase kinase 2 (CaMKK2), liver kinase B1 (LKB1), and horseradish peroxidase-conjugated secondary antibodies were obtained from Cell Signaling Technology (Beverly, MA, USA). Anti-AMPK*α* and anti-CaMKK2 antibodies were supplied from Santa Cruz Biotechnology (Santa Cruz, CA, USA). Anti-nitrotyrosine (NT) and anti-4-hydroxynonenal (4-HNE) antibodies were purchased from Millipore (Temecula, CA, USA) and Abcam (Cambridge, UK), respectively. Fluo-4-acetoxymethyl ester (Fluo-4) was obtained from Invitrogen (Carlsbad, CA, USA). 3-(4,5-dimethylthiazol-2-yl)-2,5-diphenyl-tetrazolium bromide (MTT), rhodamine 123, 2′,7′-dichlorofluorescein diacetate (DCFH-DA), CCl_4_, anti-*β*-actin antibody, and other reagents were supplied from Sigma-Aldrich (St. Louis, MO, USA).

### 2.2. Preparation of the SDT

Medicinal herbs consisting SDT were supplied from Daewon Pharmacy (Daegu, Korea) (Table 1). SDT was prepared by boiling 510.0 g of SDT in 2 L of water for 3 h. The water extracts of SDT were filtered through a 0.2 *μ*m filter paper (Nalgene, New York, NY, USA) and were lyophilized by a vacuum evaporator. The yield of lyophilized SDT was 27.08%, and the lyophilized SDT was stored at -20°C until use.

### 2.3. Chemical Profiling of SDT

To examine chemical profiles of SDT, SDT and individual standard compounds were analyzed by ultraperformance liquid chromatography (UPLC) system (Waters ACQUITY™ UPLC system, Waters Corp., Milford, MA, USA) with Waters ACQUITY™ photodiode array detector, Waters ACQUITY™ BEH C_18_ column (1.7 *μ*m, 2.1 × 100 mm), and Empower software. The samples (2 *μ*L each) dissolved in methanol were eluted using a 2% to 100% acetonitrile gradient solution containing 0.1% of formic acid. Flow rate was 0.4 mL/min. The glycyrrhizic acid was detected at the wave length of 254 nm. Calycosin-7-O-*β*-D-glucose, cinnamic acid, 6-gingerol, and 5-hydroxymethyl-2-furfural were detected at 280 nm. Paeoniflorin and ferulic acid were detected at 230 nm, decursin at 330 nm, and ginsenoside Rg1 at 203 nm, respectively. Concentrations of each compound in SDT were quantified based on peak areas and retention times.

### 2.4. Cell Culture and Treatment

HepG2 cells (a human hepatocyte-derived cell line) and HeLa cells (a human cervical cancer cell line) were purchased from American Type Culture Collection (Rockville, MD, USA) and cultured in Dulbecco's modified Eagle's medium containing 100 U/mL of penicillin, 100 *μ*g/mL of streptomycin, and 10% fetal bovine serum at 37°C with 5% CO_2_. For all experiments, the cells were grown to 80-90% confluency and starved serum for 12 h. HepG2 cells were treated with AA and iron, as described previously [9]. Briefly, cells were incubated with 10 *μ*M of AA for 12 h and then subsequently exposed to 5 *μ*M of iron for 1 h. 30-1000 *μ*g/mL of SDT was pretreated 1 h before AA treatment. For some experiments, compound C (10 *μ*M) or STO-609 (1 *μ*g/mL) was added to the medium 1 h prior to SDT treatment.

### 2.5. Cell Viability Assay

After treatments, viable cells were stained with MTT (0.5 mg/mL) for 4 h according to the previous report [21]. The relative cell viability was defined as the % of untreated control cells.

### 2.6. Preparation of Whole Cell Lysates and Immunoblot Analysis

Whole cell lysates were prepared, as previously described [22]. Briefly, the cells were lysed in radioimmunoprecipitation assay buffer containing protease inhibitor and phosphatase inhibitor cocktail (Thermo Fisher Scientific Inc., Rockford, IL, USA). After incubation for 1 h on ice, cell lysates were centrifuged at 10,000 ×*g* for 10 min. Protein concentration was quantified using a bicinchoninic acid assay kit (Thermo Fisher Scientific Inc.). Equal amounts of protein were resolved by sodium dodecyl sulfate-polyacrylamide gel electrophoresis and then transferred to nitrocellulose or polyvinylidene fluoride membranes (Amersham Biosciences, Buckinghamshire, UK). After incubating membrane with primary and secondary antibodies, immunoreactive proteins of interest were visualized by enhanced chemiluminescence detection kit (Amersham Biosciences) and image analyzing system (Imager 600, Amersham Biosciences). Equal protein loadings were verified by *β*-actin immunoblotting. Band intensity of protein interest was quantified by ImageJ software (NIH; http://imagej.nih.gov/ij).

### 2.7. Measurement of Reduced GSH

The level of reduced GSH in cell homogenates was determined by using a GSH BIOXYTECH GSH-400 kit (Oxis International Inc., Portland, OR, USA), as previously described [9]. GSH content was measured at 405 nm using a microplate reader (Infinite 200 PRO, Tecan, Männedorf, Switzerland) and was normalized by cell number.

### 2.8. Measurement of Hydrogen Peroxide (H_*2*_O_*2*_) Production

The level of H_2_O_2_ production was detected by adding the DCFH-DA [22]. After treatment, the cells were stained with 20 *μ*M of DCFH-DA for 1 h. The fluorescence emitted by dichlorofluorescein was measured at excitation/emission wavelengths of 485/530 nm using a microplate reader (Tecan).

### 2.9. Measurement of Mitochondrial Membrane Potential (MMP)

MMP was measured by using rhodamine 123, a membrane permeable cationic fluorescent dye [22]. After treatment, the cells were stained with 0.05 *μ*g/mL of rhodamine 123 for 1 h and were harvested by trypsinization. The change in MMP was detected using a flow cytometer (Partec, Münster, Germany). In each analysis, 10,000 cells were recorded.

### 2.10. Measurement of Intracellular Ca^*2*+^ Levels

To determine levels of intracellular Ca^2+^, HepG2 cells were stained with 5 *μ*M of Fluo-4 for 30 min at 37°C and then washed with PBS to remove Fluo-4 in medium. After addition of SDT (300 *μ*g/mL), the fluorescence intensities for 15 min were measured at excitation/emission wavelengths of 488/520 nm using a microplate reader (Tecan). Changes on fluorescence intensities for 10 min were calculated as area under the curve (AUC) using Graphpad prism 7 (Graphpad software, Inc., CA, USA).

### 2.11. Animal Experiments

Animal experiments were conducted according to the national regulations regarding the use and welfare of laboratory animals and were approved by the Institutional Animal Care and Use Committee in Daegu Haany University (Approval No. DHU2015-057). ICR mice at 6 weeks of age (male, 28-30 g) were supplied from Orient Bio, Inc. (Seongnam, Korea) and acclimatized for one week. Mice (N=16) were maintained with a supply of filtered pathogen-free air, provided with commercial chow (Nestle Purina PetCare Korea, Seoul, Korea) and water* ad libitum* at a temperature between 20°C and 23°C with a 12 h light/dark cycle and relative humidity of 50%. SDT dissolved in water was orally administrated to mice at the dose of 300 and 500 mg/kg/day for 4 consecutive days. One hour after the last SDT administration, mice were intraperitoneally injected 10% CCl_4_ (0.5 mL/kg) diluted with corn oil. Blood and liver tissues were collected at 24 h after CCl_4_ injection.

### 2.12. Blood Biochemistry

Alanine aminotransferase (ALT) and aspartate aminotransferase (AST) activities in serums were measured using an automated blood chemistry analyzer (Photometer 5010, Robert Riele GmbH & Co KG, Berlin, Germany).

### 2.13. Histopathology and Immunohistochemistry

Histopathology and immunohistochemistry were conducted as previously described [9]. Briefly, liver tissues were fixed with 10% neutral buffered formalin, embedded in paraffin, sectioned (3-4 *μ*m), and then stained with hematoxylin and eosin. The numbers of degenerative hepatocytes (cells/1000 hepatocytes) and the numbers of infiltrated inflammatory cells (cells/mm^2^ of hepatic parenchyma) were observed under light microscope (Model 80*i*, Nikon, Tokyo, Japan). NT and 4-HNE in hepatic tissues were stained using primary antibodies and avidin-biotin-peroxidase and peroxidase substrate kit (Vector Labs, Burlingame, CA, USA). Positive cells were defined as having NT and 4-HNE immunoreactivity intensities of > 20%. All histopathological and immunohistochemical parameters were calculated using an automated image analyzer (*i*Solution FL ver 9.1, IMT* i*-Solution Inc., Quebec, Vancouver, Canada) and the histopathologist that performed this analysis was unaware of sample details.

### 2.14. Statistical Analysis

The unpaired Student's* t*-test was used for determining the significance between two groups. One-way analysis of variance was used to assess the significance among experimental groups, followed by Tukey's honest significance difference or Dunnett's T3 as* post hoc* analysis. All data are expressed means ± standard deviation (SD) of at least three separate experiments.* P* values less than 0.05 were considered as statistical differences of significance.

## 3. Results

### 3.1. Quantification of Nine Marker Compounds in SDT

**Figure 1:**
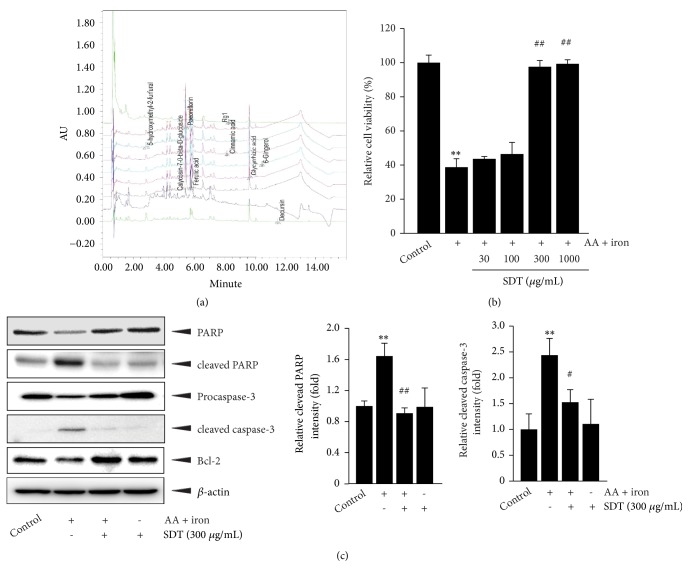
The effect of* Sipjeondaebo-tang* (SDT) on AA plus iron-mediated apoptosis. (a) UPLC chromatogram of nine marker compounds in SDT. The chromatograms were obtained at 280 nm (for 5-hydroxymethyl-2-furfural, calycosin-7-O-*β*-D-glucoside, cinnamic acid, and 6-gingerol), 230 nm (for paeoniflorin and ferulic acid), 203 nm (for ginsenoside Rg1), 254 nm (for glycyrrhizic acid), and 330 nm (for decursin), respectively. (b) Cell viability. HepG2 cells were pretreated with 30-1000 *μ*g/mL of SDT for 1 h, exposed to 10 *μ*M of AA for 12 h, and treated with 5 *μ*M iron for 1 h. Relative cell viabilities were measured by MTT assay. (c) Changes on apoptosis-related proteins. Cells were incubated with 300 *μ*g/mL of SDT for 1 h and then treated with AA plus iron, as described in panel (b). Equal protein loading was verified by *β*-actin immunoblotting (left). The intensities of cleaved forms of PARP and caspase-3 were quantified by scanning densitometry (middle and right). All values represent mean ± SD of three separated experiments; significant versus untreated control, ^∗∗^*P* < 0.01; significant versus AA plus iron, ^##^*P* < 0.01 and ^#^*P* < 0.05.

Based on the Korean pharmacopoeia, we used glycyrrhizic acid for quality control of Glycyrrhizae Radix et Rhizoma, paeoniflorin for Paeoniae Radix, ferulic acid for Cnidii Rhizoma, 5-hydroxymethyl-2-furfural for Rehmanniae Rhizoma Preparata, calycosin-7-O-*β*-D-glucose for Astragali Radix, decursin for Angelicae Gigantis Radix, ginsenoside Rg1 for Ginseng Radix, cinnamic acid for Cinnamomi Cortex, and 6-gingerol for Zingiberis Rhizoma Crudus (Ministry of Food and Drug Safety, https://www.mfds.go.kr/herbmed and https://ezdrug.mfds.go.kr/, accessed 17 Mar 2106, in Korean). Before investigating the hepatoprotective effects of SDT, we quantified nine chemical markers in SDT via UPLC under optimized conditions (Figure S1). The SDT used in the present study contained 253.62 ± 4.43 ppm of glycyrrhizic acid, 147.07 ± 1.82 ppm of paeoniflorin, 49.72 ± 1.7 ppm of ferulic acid, 32.31 ± 1.62 ppm of 5-hydroxymethyl-2-furfural, 22.97 ± 0.83 ppm of calycosin-7-O-*β*-D-glucose, 4.74 ± 0.72 ppm of decursin, 2.62 ± 0.03 ppm of ginsenoside Rg1, 1.82 ± 0.44 ppm of cinnamic acid, and 0.87 ± 0.05 ppm of 6-gingerol (Figure 1(a)).

### 3.2. SDT Inhibits AA Plus Iron-Mediated Apoptosis

We and others have reported that treatment with AA plus iron induces oxidative stress-mediated apoptosis in HepG2 cells [3, 8, 9]. To examine whether SDT protects cells from oxidative stress-mediated cytotoxicity, HepG2 cells were pretreated with 30-1000 *μ*g/mL of SDT for 1 h and subsequently exposed to AA (10 *μ*M, 12 h) and iron (5 *μ*M, 1 h); then, cell viability was determined using the MTT assay. As expected, treatment with AA plus iron significantly decreased cell viability (38.70 ± 4.99%) compared with the untreated control. However, pretreatment with 300 and 1000 *μ*g/mL of SDT completely prevented the reduction in cell viability induced by AA plus iron, while 30 and 100 *μ*g/mL of SDT pretreatment did not (Figure 1(b)). Treatment with SDT alone at up to 1000 *μ*g/mL did not change the viability of HepG2 cells (Figure S2(a)).* tert*-butyl hydroperoxide is an organic peroxide that provokes oxidative stress in HepG2 cells [22]. In parallel with the results of AA plus iron treatment, SDT pretreatment (100 and 300 *μ*g/mL) significantly prevented* tert*-butyl hydroperoxide-mediated cytotoxicity in HepG2 cells (Figure S2(b)). Because the maximal preventive effect against AA plus iron-mediated cytotoxicity was observed with pretreatment above 300 *μ*g/mL of SDT, 300 *μ*g/mL of SDT was used in subsequent experiments. Next, we monitored expression levels of apoptosis-related proteins by immunoblot analysis. Pretreatment with SDT (300 *μ*g/mL) in the presence of AA plus iron decreased the expression of the cleaved forms of PARP and caspase-3 and increased the expression of Bcl-2 (Figure 1(c)). These results indicate that SDT can prevent oxidative stress-mediated apoptosis in HepG2 cells.

### 3.3. SDT Inhibits AA Plus Iron-Mediated Oxidative Stress and Mitochondrial Dysfunction

**Figure 2:**
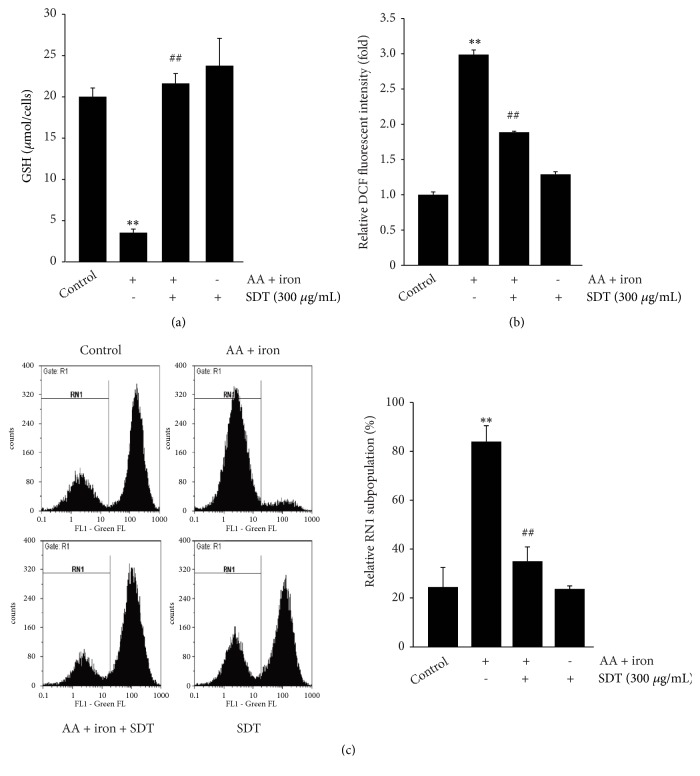
The effect of SDT on AA plus iron-mediated oxidative stress and mitochondrial dysfunction. (a) Cellular GSH contents. HepG2 cells were treated SDT (300 *μ*g/mL), AA (10 *μ*M), and iron (5 *μ*M), as described in Figure 1(b). Reduced GSH contents were estimated in cell homogenates. (b) H_2_O_2_ production. Treated cells were stained with 20 *μ*M DCFH-DA for 30 min, and intracellular levels of H_2_O_2_ were measured using dichlorofluorescein fluorescence intensity. (c) MMP. Fluorescence intensity of rhodamine 123-stained cells was monitored using a flow cytometer (left). Cell subpopulation with low rhodamine 123 fluorescence intensity (RN1 fraction) was represented as percentages of total cell analyzed (right). All values represent mean ± SD of three separated experiments; significant versus untreated control, ^∗∗^*P* < 0.01; significant versus AA plus iron, ^##^*P* < 0.01.

To investigate whether the reduction in oxidative stress conferred by SDT contributes to the protection of cells against AA plus iron, we measured intracellular concentrations of reduced GSH, the major endogenous antioxidant. Treatment with AA plus iron depleted GSH levels in HepG2 cells. However, pretreatment with SDT (300 *μ*g/mL) significantly prevented GSH depletion by AA plus iron (Figure 2(a)). Next, we monitored intracellular levels of H_2_O_2_ after staining cells with DCFH-DA. Treatment with AA plus iron increased H_2_O_2_ production, whereas SDT pretreatment significantly reduced H_2_O_2_ production (Figure 2(b)). Intracellular levels of GSH and H_2_O_2_ did not differ between the treatment with SDT alone and untreated control. Because impairment of mitochondrial respiration results in intracellular ROS accumulation, we further examined whether SDT could restore MMP. From the flow cytometry results, AA plus iron increased the subpopulation with low rhodamine 123 fluorescence intensity (RN1 subpopulation), and SDT pretreatment significantly inhibited the increase in the RN1 subpopulation. SDT alone had no effect on the RN1 subpopulation (Figure 2(c)). These results suggest that SDT protects cells by reducing oxidative stress and restores mitochondrial integrity.

### 3.4. AMPK Activation Is Essential for SDT-Mediated Cytoprotection

**Figure 3:**
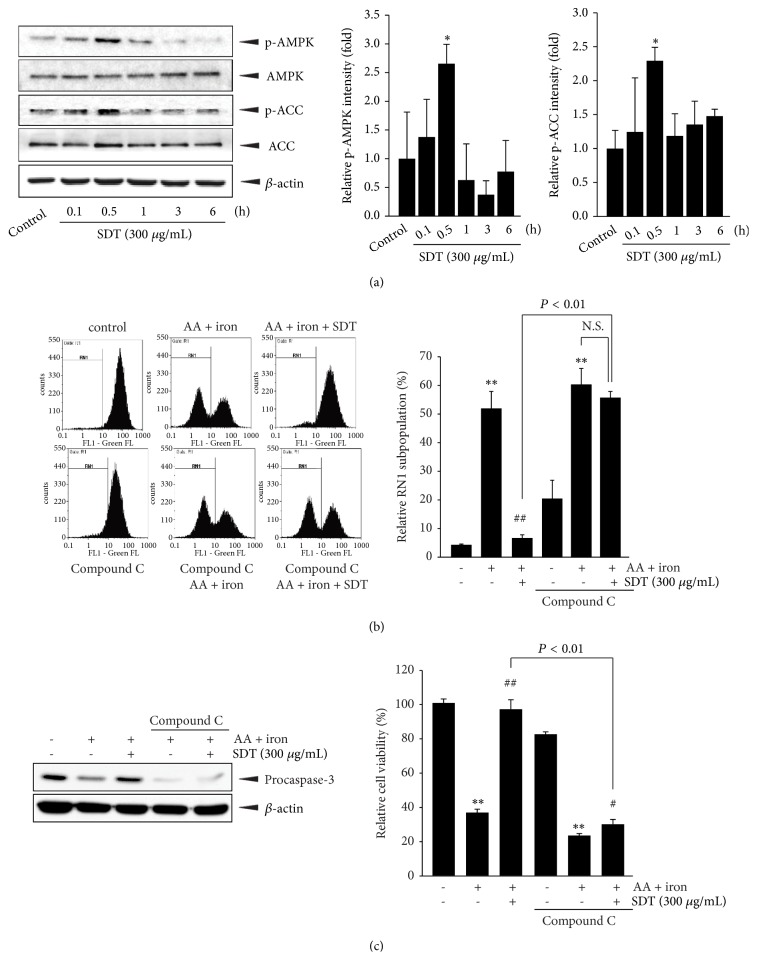
The effect of SDT on AMPK activation. (a) AMPK phosphorylation. Immunoblot analyses were conducted using HepG2 cell lysates that had been treated with SDT (300 *μ*g/mL) for indicated time periods. Equal protein loading was verified by *β*-actin immunoblotting (left). Relative levels of phosphorylated AMPK (middle) and phosphorylated ACC (right) were quantified by scanning densitometry. (b) Effect of compound C on SDT-mediated mitochondrial protection. 10 *μ*M of compound C-pretreated HepG2 cells (1 h) were exposed to SDT, AA, and iron. (c) Effect of compound C on SDT-mediated cytoprotection. Expression of procaspase 3 (left) and relative cell viability (right) were monitored by immunoblot and MTT analysis, respectively. All values represent mean ± SD of three separated experiments; significant versus untreated control, ^∗∗^*P* < 0.01 and ^∗^*P* < 0.05; significant versus AA plus iron, ^##^*P* < 0.01 and ^#^*P* < 0.05; NS, not significant.

Because we and others have reported several medicinal herbs and natural products that prevent oxidative stress via AMPK activation [4, 7–10], we first monitored the effect of SDT (300 *μ*g/mL) on the temporal responses of AMPK and ACC (a representative downstream substrate of AMPK) phosphorylation. Based on immunoblot results, maximal increases in AMPK and ACC phosphorylation were observed at 0.5 h after SDT treatment, after which the levels gradually decreased. SDT treatment did not change AMPK and ACC expression (Figure 3(a)). To explore whether AMPK activation by SDT contributes to the protection of mitochondria and cells from AA plus iron, HepG2 cells were pretreated with 10 *μ*M of compound C (a chemical inhibitor of AMPK), and exposed to SDT, AA, and iron. Pretreatment with compound C alone in HepG2 cells tended to increase the RN1 subpopulation. In addition, compound C inhibited the protective effect of SDT on AA plus iron-mediated mitochondrial membrane leakage (Figure 3(b)). Moreover, restorations of procaspase-3 expression and cell viability by SDT were blocked by pretreatment with compound C (Figure 3(c)). These results imply that SDT-induced AMPK activation is responsible for protecting cells and mitochondria.

### 3.5. CaMKK2 Is an Upstream Kinase of SDT-Mediated AMPK Activation

**Figure 4:**
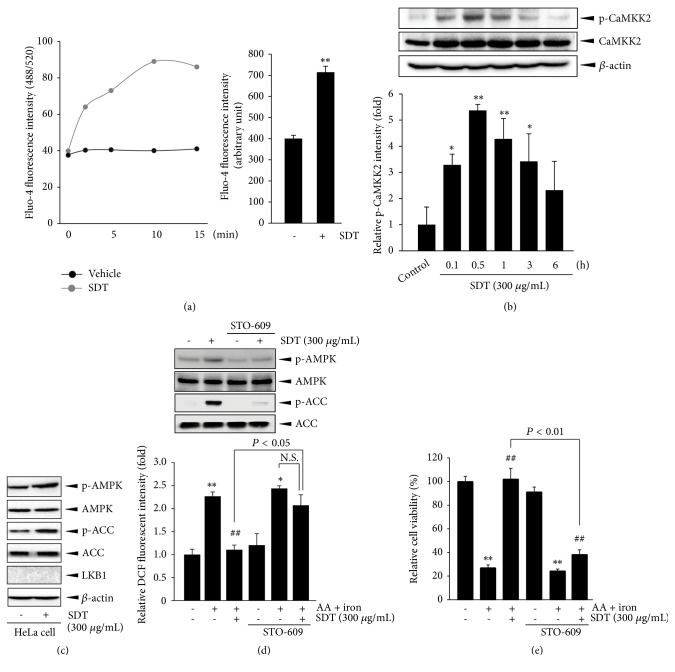
The effect of SDT on CaMKK2 activation. (a) Effect of SDT on intracellular Ca^2+^ level. Fluo-4 stained HepG2 cells were incubated with SDT (300 *μ*g/mL), and fluorescence intensity for 15 min was monitored (left). Changes in fluorescence intensity for 10 min were calculated as AUC (right). (b) CaMKK2 phosphorylation by SDT. CaMKK2 phosphorylation was determined by using HepG2 cell lysates that had been treated with SDT (300 *μ*g/mL) for indicated times. Equal protein loading was verified by *β*-actin immunoblotting (upper). Intensity of phosphorylated CaMKK2 was quantified by scanning densitometry (lower). (c) Effect of SDT on HeLa cells. HeLa cells were treated with 300 *μ*g/mL SDT for 0.5 h, and AMPK and ACC phosphorylation were determined by immunoblot analysis. Phenotype of HeLa cells was verified by LKB1 immunoblotting. (d) Effect of STO-609 on the inhibition of AA plus iron-mediated H_2_O_2_ production by SDT. SDT-dependent phosphorylation of AMPK (upper) and reduction of H_2_O_2_ production (lower) were monitored in STO-609 (1 *μ*g/mL, 1 h) pretreated cells. (e) Effect of STO-609 on SDT-mediated cytoprotection. The relative cell viability was determined using MTT assay. All values represent mean ± SD of three separated experiments; significant versus untreated control, ^∗∗^*P* < 0.01 and ^∗^*P* < 0.05; significant versus AA plus iron, ^##^*P* < 0.01; NS, not significant.

To identify the upstream kinase of SDT-mediated AMPK activation, HepG2 cells were stained with 5 *μ*M of Fluo-4, and intracellular levels of Ca^2+^ were measured. Treatment with SDT (300 *μ*g/mL) rapidly increased the Fluo-4 fluorescence intensity, which peaked after 10 min and was sustained for 15 min (Figure 4(a), left). When the changes in fluorescence intensity for 10 min were calculated as AUC, SDT treatment significantly increased intracellular Ca^2+^ levels compared with the untreated control (Figure 4(a), right). In addition, treatment with SDT significantly increased CaMKK2 phosphorylation (Figure 4(b)), and the maximum level of CaMKK2 phosphorylation was detected at 0.5 h, concurrent with the maximum AMPK phosphorylation (Figure 3(a)). It has been known that LKB1, another upstream kinase of AMPK, is deficient in HeLa cells [23]. When HeLa cells were treated with SDT, SDT phosphorylated AMPK and ACC (Figure 4(c)). In addition, pretreatment with STO-609 (1 *μ*g/mL; a representative CaMKK2 inhibitor) decreased SDT-dependent AMPK and ACC phosphorylation (Figure 4(d), upper). Moreover, STO-609 blocked the effects of SDT on AA plus iron-mediated H_2_O_2_ production (Figure 4(d), lower) and cytotoxicity (Figure 4(e)). These results suggest that SDT activates AMPK via CaMKK2 phosphorylation.

### 3.6. SDT Protects CCl_*4*_-Induced Liver Injury in Mice

**Figure 5:**
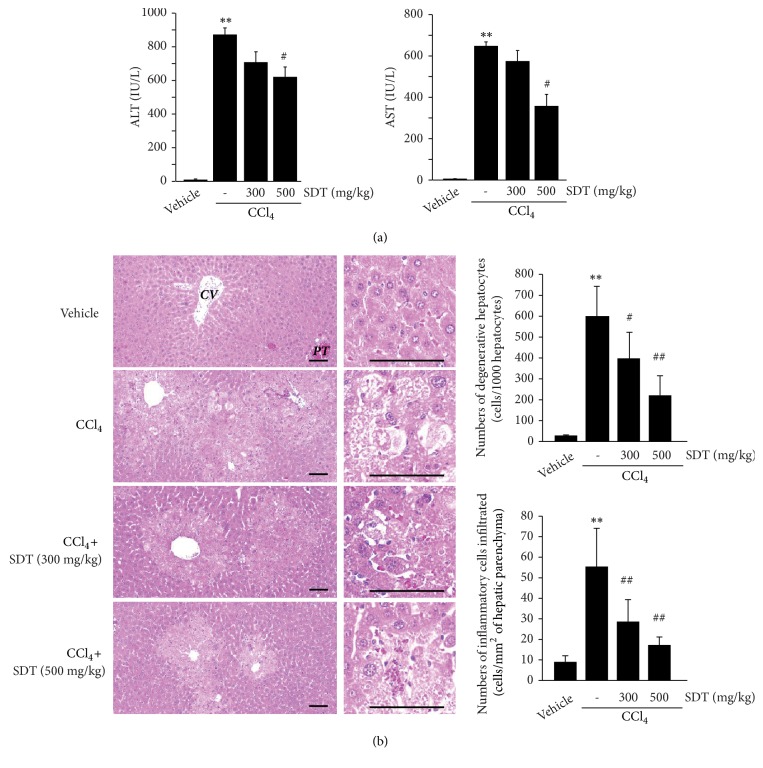
The effect of SDT on CCl_4_-induced acute liver injury. (a) ALT and AST activities. Mice were orally administered SDT (300 or 500 mg/kg) for 4 days. One hour after last SDT administration, mice were intraperitoneally injected CCl_4_ (0.5 mL/kg). Serum ALT (left) and AST (right) activities were detected 24 h after CCl_4_ injection. (b) The representative histological profiles of the liver tissues. Scale bars indicate 120 *μ*m (left). The numbers of degenerative hepatocytes (upper right) and infiltrated inflammatory cells (lower right) were quantified using an automated image analyzer. All values represent mean ± SD of four mice (for (a)) or eight historical fields from four mice (for (b)); significant versus vehicle-treated group, ^∗∗^*P* < 0.01; significant versus CCl_4_-treated group, ^##^*P* < 0.01 and ^#^*P* < 0.05; CV, central vein; PT, portal triad.

**Figure 6:**
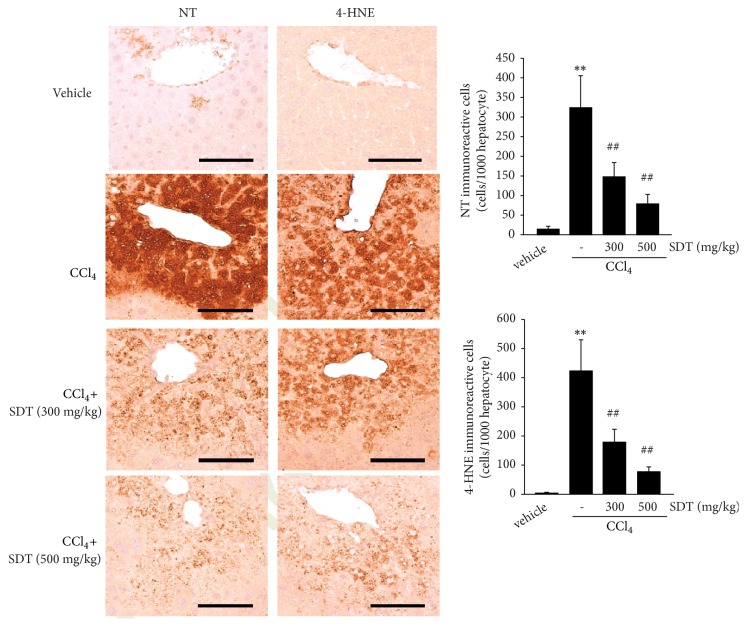
The effect of SDT on CCl_4_-induced oxidative stress in hepatic tissues. Immunohistochemical analyses in liver tissues were conducted using anti-NT or anti-4-HNE antibody (left). The numbers of NT- (upper right) and 4-HNE-positive cells (lower right) were measured using an automated image analyzer. Scale bars indicate 120 *μ*m. All values represent mean ± SD of eight historical fields from four mice; significant versus vehicle-treated group, ^∗∗^*P* < 0.01; significant versus CCl_4_-treated group, ^##^*P* < 0.01.

To expand on our finding that SDT protects cells from oxidative stress-mediated apoptosis* in vitro*, 300 or 500 mg/kg of SDT was administered to ICR mice for 4 days, after which CCl_4_ was injected to induce oxidative stress-mediated liver injury. The activities of ALT and AST, which are serum biochemical markers of liver injury, significantly increased at 1 day after CCl_4_ injection compared with vehicle-injected mice. However, oral administration of SDT for 4 consecutive days tended to reduce serum ALT and AST activities in a dose-dependent manner, and a significant difference was only observed between the 500 mg/kg of SDT and CCl_4_ groups (Figure 5(a)). Histomorphometric analysis of hematoxylin and eosin-stained hepatic tissues indicated that the number of degenerative hepatocytes showing acute cellular swelling, severe fatty changes, and eosinophilic necrosis was significantly increased after CCl_4_ injection, and SDT administration prevented hepatocyte degeneration in a dose-dependent manner. In addition, SDT significantly reduced infiltration of inflammatory cells in hepatic parenchyma by CCl_4_ (Figure 5(b)). To examine whether SDT protects the liver from CCl_4_-induced toxicity by reducing oxidative stress* in vivo*, liver tissues were stained with NT (a marker of nitrosative stress) and 4-HNE (a marker of lipid peroxidation). SDT administration significantly reduced the numbers of NT- and 4-HNE-immunoreactive cells in hepatic tissues in a dose-dependent manner (Figure 6). These results indicate that SDT can protect the liver from oxidative stress* in vivo*.

## 4. Discussion

Use of herbal medicines has become common worldwide due to their multiple therapeutic activities and few side effects [24]. SDT is one of the most well-known herbal prescriptions for treating weakness after illness (e.g., anorexia, fatigue, and anemia) in Korea, China, and Japan [11, 12]. Although several studies have reported the effects of SDT against oxidative stress in the liver [15, 16], the associated molecular mechanisms by which SDT prevents oxidative stress have not been fully elucidated. Before testing the antioxidant potential of SDT, we conducted a UPLC analysis to assess the quality of SDT and observed chemical markers representing nine medicinal herbs effectively solubilized in the SDT water extract. Of the nine marker compounds, glycyrrhizic acid, ferulic acid, cinnamic acid, 6-gingerol, 5-hydroxymethyl-2-furfural, paeoniflorin, and ginsenoside Rg1 have been reported to reduce oxidative stress in experimental hepatic disorders [25–31]. However, the concentrations of the nine marker compounds were too low to explain the potent hepatoprotective effects of SDT. Thus, these compounds in combination with unidentified compounds might confer protection of the liver from oxidative stress; additional studies are required to identify the major contributors to the hepatoprotective effect of SDT.

Based on reports that AA in the presence of iron synergistically accelerates oxidative stress-mediated apoptosis [3], we used an AA plus iron exposure model in HepG2 cells to investigate the cytoprotective effect of SDT and elucidate the molecular mechanism* in vitro*. Here, we showed that pretreatment with 300 or 1000 *μ*g/mL SDT completely prevented AA plus iron-mediated cytotoxicity. Moreover, SDT inhibited the GSH depletion, H_2_O_2_ production, and mitochondrial membrane impairment induced by AA plus iron. SDT also attenuated the reduction of Bcl-2, an antiapoptotic protein and decreased the activation of apoptosis executors (e.g., procaspase-3 and PARP cleavage). Overall, these results suggest that SDT has hepatoprotective effects against AA plus iron-mediated apoptosis by reducing oxidative stress.

We previously reported that eupatilin, tryptanthrin, isorhamnetin,* Paeonia obovata*, and* Buddleja officinalis* protect cells from the effects of AA plus iron via AMPK activation [4, 7–9, 32]. AMPK is regarded as a master regulator of cellular homeostasis related to oxidative and metabolic stress [5]. In our previous study, blockage of AMPK activation by a chemical inhibitor (e.g., compound C) or dominant negative mutant of AMPK failed to reduce the H_2_O_2_ generation, mitochondrial membrane impairment, and cytotoxicity induced by AA plus iron [4, 7, 9, 32]. Similarly, the present results showed that SDT increased AMPK and ACC phosphorylation. Moreover, SDT-mediated protection of the mitochondrial membrane and cells was completely blocked by pretreatment with compound C. These results provide evidence that SDT acts as a hepatoprotective herbal medicine via AMPK activation.

CaMKK2 (also known as CaMKK*β*), LKB1, and TGF-*β*-activated kinase 1 have been suggested to phosphorylate directly within the activation loop (i.e., Thr172) of the catalytic *α* subunit [5, 6, 33]. Different upstream kinases seem to phosphorylate context-dependently AMPK. Microeconomic metabolic stress phosphorylates AMPK via nucleotide-dependent LKB1 activation, while increases in intracellular Ca^2+^ by external stimuli (i.e., macroeconomic metabolic stress) lead to CaMKK2 activation, which phosphorylates Thr172 and activates AMPK in a nucleotide-independent manner [34–36]. Tissue distribution studies of LKB1 reveal that LKB1 mediates the AMPK phosphorylation in every tissue. However, CaMKK2-dependent AMPK phosphorylation appears nearly in neurons, T cells, and liver [37, 38]. Moreover, systemic and liver specific deletion of CaMKK2 ameliorates diet-induced metabolic stress [38, 39]. In the present study, we showed that SDT increased intracellular Ca^2+^ levels and CaMKK2 phosphorylation. In addition, SDT could phosphorylate AMPK in HeLa cells (representative LKB1-deficient cells). When cells were pretreated with STO-609, a selective CaMKK2 inhibitor, SDT-mediated AMPK, and ACC phosphorylation decreased. Thus, LKB1 does not appear to be an important upstream kinase for SDT-mediated AMPK phosphorylation at least in our experimental system. Moreover, pretreatment with STO-609 reduced the cytoprotective ability of SDT and sustained H_2_O_2_ production against AA plus iron. These results suggest that CaMKK2 is an upstream signaling molecule of SDT-dependent AMPK activation.

We previously found that eupatilin increased Sestrin2-mediated AMPK phosphorylation and reduced oxidative stress via autophagy induction [8]. In addition, downstream p38 mitogen-activated protein kinase (p38 MAPK) activation and GSK-3*β* inhibition are also involved in AMPK-mediated cytoprotection against AA plus iron [7, 9]. To explore the role of autophagy and p38 MAPK in SDT-mediated cytoprotection, HepG2 cells were additionally pretreated with chemical inhibitors of autophagy (bafilomycin A1 and 3-methyladenine) or p38 MAPK (SB203580). However, the cytoprotective ability of SDT against AA plus iron was sustained in the presence of autophagy inhibitors (Figures S3(a) and S3(b)). Interestingly, our preliminary experiments using chemical inhibitors showed that the cytoprotective effect of SDT partly reduced by pretreatment with SB203580 (Figure S3(c)). Moreover, SDT-mediated cytoprotection was completely inhibited by Akt inhibitor, LY294002 (Figure S3(d)). In parallel with previous observation [9], pretreatment with a GSK-3 inhibitor (SB216763) partly (but significantly) alleviated AA plus iron-mediated cytotoxicity (data not shown). Studies have suggested that AMPK increases inactive phosphorylation of GSK-3*β* through the activation of the phosphatidylinositol-3-kinase/Akt signaling pathway [40–42]. Therefore, SDT appears to protect cells from oxidative stress in an autophagy-independent and Akt/GSK-3*β*/p38 MAPK-dependent manner. Further studies are needed to explore the relationship between AMPK and Akt in SDT treatment.

CCl_4_ is one of the most commonly used hepatotoxins to examine the hepatoprotective effects of beneficial compounds* in vivo *[4, 43]. Metabolic conversion of CCl_4_ in liver produces trichloromethyl and trichloromethyl peroxy radicals, which cause the changes in membrane permeability and provoke oxidative stress-mediated hepatocyte damage in zone 3 of the liver [4, 43, 44]. In parallel with previous studies [4, 16], the present results showed that a single CCl_4_ injection increased ALT and AST activity in mice serum, indicative of liver damage. Although SDT tended to decrease the levels of serum markers of hepatotoxicity in a dose-dependent manner, the reductions in ALT and AST activity were statistically significant only in mice administered with 500 mg/kg of SDT. Histopathological analysis of hepatic tissues revealed increases in degenerated hepatocytes around the central veins after CCl_4_ injection. Moreover, inflammatory cells infiltrated into the hepatic parenchyma by CCl_4_. However, administration of SDT for 4 consecutive days dose-dependently attenuated these changes. Reactive nitrogen species mainly produced via oxidative burst of infiltrated inflammatory cells rapidly penetrate neighboring hepatic parenchyma, accelerating dysfunction of biomolecules by covalent modification of reactive nitrogen species. In addition, free radicals produced by CCl_4_ cause lipid peroxidation and decrease membrane integrity. Thus, immunostaining against nitrated tyrosine and 4-hydroxynonenal have been commonly used to monitor oxidative stress in the liver [4, 7, 9]. Our results showed that SDT significantly reduced the increases in NT- and 4-HNE-positive cells induced by CCl_4_. These results provide evidence that SDT inhibits oxidative (nitrative) stress, protecting hepatocytes against the acute injuries caused by CCl_4_.

In conclusion, the results of present study indicate that SDT blocks AA plus iron-mediated toxicity by reducing GSH depletion, intracellular H_2_O_2_ production, and mitochondrial impairment. In addition, CaMKK2-dependent AMPK phosphorylation has an essential role in the cytoprotective effects of SDT. Meanwhile, histopathological and immunohistochemical examination revealed that SDT can ameliorate CCl_4_-mediated acute hepatotoxicity by reducing oxidative stress* in vivo*. These findings suggest that SDT is a beneficial hepatoprotective herbal medicine against oxidative stress that act via activation of the CaMKK2-AMPK signaling pathway.

## Figures and Tables

**Table 1:** Herbal constitution of *Sipjeondaebo-tang* (SDT).

Name of medical herb	Contents in the prescription (g)
Angelicae Gigantis Radix	45.0
Cnidii Rhizoma	45.0
Paeoniae Radix	45.0
Rehmanniae Rhizoma Preparata	45.0
Ginseng Radix	45.0
Atractylodis Rhizoma Alba	45.0
Poria Sclerotium	45.0
Glycyrrhizae Radix et Rhizoma	45.0
Astragali Radix	37.5
Cinnamomi Cortex	37.5
Zingiberis Rhizoma Crudus	37.5
Zizyphi Fructus	37.5

## Data Availability

The data used to support the findings of this study are available from the corresponding author upon request.

## Abbreviations

AA: Arachidonic acid
ACC: Acetyl-CoA carboxylase
ALT: Alanine aminotransferase
AMPK: AMP-activated protein kinase
AST: Aspartate aminotransferase
AUC: Area under the curve
Bcl-2: B cell lymphoma-2
CaMKK2: Ca^2+^/calmodulin-dependent protein kinase kinase 2
CCl_4_: Carbon tetrachloride
DCFH-DA: 2′,7′-Dichlorofluorescein diacetate
Fluo-4: Fluo-4-acetoxymethyl ester
GSH: Glutathione
GSK-3*β*: Glycogen synthase-3*β*
4-HNE: 4-Hydroxynonenal
H_2_O_2_: Hydrogen peroxide
LKB1: Liver kinase B1
MMP: Mitochondrial membrane potential
MTT: 3-(4,5-Dimethylthiazol-2-yl)-2,5-diphenyl-tetrazolium bromide
NT: Nitrotyrosine
p38 MAPK: p38 mitogen-activated protein kinase
PARP: Poly(ADP-ribose)polymerase
ROS: Reactive oxygen species
SD: Standard deviation
SDT: * Sipjeondaebo-tang*
UPLC: Ultraperformance liquid chromatography.

## Supplementary Materials

Supplementary Materials **Figure S1:** representative UPLC chromatogram of nine marker compounds.** Figure S2:** cell viability assay.** Figure S3:** effect of some chemical inhibitors on SDT-mediated cytoprotection.* (Supplementary Information)*
