# Supplementary material for: Sipjeondaebo-tang Alleviates Oxidative Stress-Mediated Liver Injury through Activation of the CaMKK2-AMPK Signaling Pathway
Source: Evid Based Complement Alternat Med. 2018 Nov 6;2018:8609285. doi: 10.1155/2018/8609285 (PMC6247439; doi:10.1155/2018/8609285)
Supplement: Supplementary Materials — Figure S1: representative UPLC chromatogram of nine marker compounds. Figure S2: cell viability assay. Figure S3: effect of some chemical inhibitors on SDT-mediated cytoprotection. (Supplementary Information) [file 8609285.f1.docx]

*Supplementary information*

***Sipjeondaebo-tang* alleviates oxidative stress-mediated liver injury through activation of the CaMKK2-AMPK signaling pathway**

Sang Mi Park^1,#^, Sung Woo Kim^2,#^, Eun Hye Jung^1,#^, Hae Li Ko^1^, Chae Kwang Im^2^, Jong Rok Lee^3^, Sung Hui Byun^1^, Sae Kwang Ku^4^, Sang Chan Kim^1^, Chung A Park^5^, Kwang Joong Kim^2,*^, Il Je Cho ^1,**^

^1^Department of Herbal Formulation, College of Korean Medicine, ^2^Department of Physiology, College of Korean Medicine, ^3^Department of Pharmaceutical Engineering, College of Bio-technology ^4^Department of Histology and Anatomy, College of Korean Medicine, ^5^Department of Internal Medicine, College of Korean Medicine, Daegu Haany University, Gyeongsan 38610, Republic of Korea

^#^These authors contributed equally to this work.

Corresponding author:

^*^Kwang Joong Kim, K.M.D., Ph.D., Department of Physiology, College of Korean Medicine, Daegu Haany University, Gyeongsan, Gyeongsangbuk-do 38610, Republic of Korea. Tel: +8253-819-1877, E-mail: kwangj@dhu.ac.kr

^**^Il Je Cho, Ph.D., Department of Herbal Formulation, College of Korean Medicine, Daegu Haany University, Gyeongsan, Gyeongsangbuk-do 38610, Republic of Korea. Tel: +8253-819-1295, Fax: +8253-819-1860, E-mail: skek023@dhu.ac.kr

*Supplementary materials*

Glycyrrhizic acid was obtained from Wako Pure Chemical Industries (Osaka, Japan). Ferulic acid and calycosin-7-O-β-D-glucoside were purchased from Wuhan ChemFaces Biochemical Co., Ltd. (Wuhan, China). Bafilomycin A1 and LY294004 were purchased from Santa Cruz Biotechnology (Santa Cruz, CA, USA) and Calbiochem (San Diego, CA, USA), respectively. Cinnamic acid, 6-gingerol, 5-hydroxymethyl-2-furfural, paeoniflorin, decursin, ginsenoside Rg1, 3-methyladenine, SB203580, and *tert*-butyl hydroperoxide were supplied from Sigma-Aldrich (St. Louis, MO, USA).

*tert-butyl hydroperoxide-mediated cytotoxicity*

SDT-pretreated HepG2 cells were subsequently exposed to 150 μM *tert*-butyl hydroperoxide for 12 h. The cell viability was determined by adding MTT.

**Figure S1.** Representative UPLC chromatogram of nine marker compounds. UPLC was conducted, as described in materials and methods section.

**Figure S2.** Cell viability assay. (a) Effect of SDT on HepG2 cell viability. HepG2 cells were treated with 30-1000 μg/mL SDT for 12 h. (b) Effect of SDT on *tert*-butyl hydroperoxide-induced cytotoxicity. HepG2 cells were pretreated with 100 or 300 mg/mL of SDT for 1 h, and further exposed to 150 μM of *tert*-butyl hydroperoxide for 12 h. All values represent mean ± S.D. of three separated experiments; significant versus untreated control, ^**^*P* < 0.01; significant versus *tert*-butyl hydroperoxide, ^##^*P* < 0.01; *t*BHP, *tert*-butyl hydroperoxide; N.S., not significant.

**Figure S3.** Effect of some chemical inhibitors on SDT-mediated cytoprotection. HepG2 cells were pretreated with bafilomycin A1 (2 μM)(a), 3-methyladenine (5 mM)(b), SB203580 (20 μM)(c), or LY294002 (10 μM)(d) for 1 h, and then treated with SDT, AA, and iron, as described in figure 1B. Relative cell viability was determined by MTT assay. All values represent mean ± S.D. of three separated experiments; significant versus untreated control, ^**^*P* < 0.01; significant versus AA plus iron, ^##^*P* < 0.01, ^#^*P* < 0.05; N.S., not significant.
